# Concomitant Liver Biopsy During Bariatric Surgery: No Increase in Major Complications or Readmissions but Associated with Higher Transfusion Requirements - An Analysis of the MBSAQIP Database

**DOI:** 10.1007/s11695-025-08267-9

**Published:** 2025-09-25

**Authors:** Juan S. Barajas-Gamboa, Kayanne Khoury, Valentin Mocanu, Mélissa V. Wills, Thomas H. Shin, Gustavo Romero-Velez, Matthew Allemang, Andrew T. Strong, Salvador Navarrete, Ricard Corcelles, A. Daniel Guerron, John Rodriguez, Matthew Kroh, Jerry T. Dang

**Affiliations:** 1 Digestive Disease Institute, Department of General Surgery, Cleveland Clinic Abu Dhabi, Abu Dhabi, United Arab Emirates; 2 The Royal College of Surgeons in Ireland – Medical University of Bahrain, Muharraq, Bahrain; 3 Digestive Disease Institute, Department of General Surgery, Cleveland Clinic, Cleveland, OH USA; 4 Department of General Surgery, University of Virginia, Charlottesville, VA USA; 5 Cleveland Clinic Lerner College of Medicine of Case Western Reserve University, Cleveland, OH USA

**Keywords:** Bariatric surgery, Liver biopsy, Surgical complications, Patient safety, MBSAQIP, Obesity

## Abstract

**Introduction:**

Concomitant liver biopsy during bariatric surgery has gained interest for diagnostic and research purposes, particularly in studying obesity-related liver disease. However, comprehensive data on safety remains limited using recent national databases.

**Methods:**

A retrospective analysis of the MBSAQIP database from 2020 to 2022 was conducted. Patients undergoing primary Roux-en-Y gastric bypass or sleeve gastrectomy with or without concomitant liver biopsy were included. Primary outcomes were 30-day major complications (Clavien-Dindo grade III and IV) and mortality. Separate multivariable analyses were performed for bleeding-related complications including postoperative bleeding, transfusion requirements, and reoperation. Multivariable logistic regression analysis determined if concomitant liver biopsy independently predicted adverse outcomes.

**Results:**

Of 511,981 patients, 30,819 (6.02%) underwent concomitant liver biopsy. Biopsy patients had higher rates of diabetes (28.25% vs 22.65%, *p* < 0.001) and hyperlipidemia (25.20% vs 22.14%, *p* < 0.001). Mean operative time was longer in the biopsy group (96.10 vs 83.59 min, *p* < 0.001). Unadjusted analysis showed higher rates of major complications (Clavien-Dindo grade III and IV) (3.15% vs 2.70%, *p* < 0.001) and 30-day readmission (3.32% vs 2.86%, *p* < 0.001) in the biopsy group. However, on multivariable analysis, concomitant liver biopsy was not independently predictive of major complications (OR 0.91, 95%CI 0.79–1.04, *p* = 0.164) or 30-day readmission (OR 1.06, 95%CI 0.94–1.21, *p* = 0.344). Separate multivariable analyses for bleeding-related complications revealed that concomitant liver biopsy was independently associated with increased postoperative bleeding (OR 1.203, 95%CI 1.018–1.422, *p* = 0.030) and transfusion requirements (OR 1.171, 95%CI 1.028–1.334, *p* = 0.018), but not with reoperation (OR 1.016, 95%CI 0.908–1.138, *p* = 0.783).

**Conclusion:**

After adjusting for patient factors, concomitant liver biopsy during bariatric surgery does not independently increase the risk of major complications (Clavien-Dindo grade III and IV), readmission, or mortality. However, it is associated with increased bleeding and transfusion complications. These findings support that liver biopsy can be performed during bariatric surgery, though clinicians should be prepared for increased bleeding-related risks and blood product utilization.

## Introduction

Morbid obesity has emerged as one of the strongest contributors to the development and progression of Metabolic dysfunction–associated steatotic liver disease (MASLD) [1–4]. Recent epidemiological data from 2020 demonstrates the significant burden of MASLD, with a worldwide prevalence of 32.16% [5] and a prevalence of 24% in the United States [6]. The prevalence of MASLD is substantially higher in bariatric surgery candidates, ranging from 75–95%, with 20–35% showing advanced forms including steatohepatitis and fibrosis. Primary risk factors include insulin resistance, diabetes, dyslipidemia, and visceral adiposity, with disease severity correlating with both BMI magnitude and obesity duration. Despite this high prevalence, MASLD often remains clinically silent even in advanced stages [5, 6] leading many to pursue intra-operative liver biopsy at the time of metabolic surgery to better characterize MASLD.

Of particular concern is the potential progression to Metabolic dysfunction-associated steatohepatitis (MASH) [7], which significantly increases the risk of cryptogenic cirrhosis and hepatocellular carcinoma [8, 9]. Projections indicate that MASH will become the leading indication for liver transplantation in the United States, substantially increasing healthcare resource utilization [10]. The financial implications are considerable, with incremental costs ranging from $32,930 to $57,544 per patient per year [11], emphasizing the critical importance of early detection and prevention of disease progression. For eligible patients meeting established criteria [12], bariatric surgery (BS) has demonstrated remarkable effectiveness in managing morbid obesity, achieving not only significant weight loss and metabolic improvement [13] but also substantial histological improvement in MASLD [14, 15].

While previous studies have examined the safety of concomitant liver biopsy during bariatric surgery using earlier the Metabolic and Bariatric Surgery Accreditation and Quality Improvement Program (MBSAQIP) datasets [16], there is a need for updated analysis reflecting contemporary practice patterns. Our study offers several important advances beyond previous analyses: 1) a more comprehensive multivariable model controlling for a wider range of potential confounders; 2) separate detailed analyses of major complications and readmissions as well as specific bleeding-related outcomes including transfusion requirements; 3) utilization of enhanced variables and more consistent coding in the 2020–2022 MBSAQIP dataset; and 4) insights into practice patterns following updated MASLD guidelines and evolving surgical techniques. Recent advances in bariatric surgical techniques, increased awareness of MASLD among surgeons, and evolving clinical practice guidelines may have influenced both the frequency and outcomes of intraoperative liver biopsies [17].

The need to understand and monitor liver disease progression in this high-risk population has driven efforts to develop reliable diagnostic tools. While non-invasive methods such as FibroScan and liver biomarkers have been explored, recent evidence suggests these tools lack the sensitivity needed to detect subtle forms of liver disease [18]. Liver biopsy remains the gold standard for MASLD diagnosis [19] and serves a crucial role in resolving diagnostic uncertainties. Furthermore, it provides invaluable prognostic information for MASH patients, enabling the identification of advanced fibrosis cases before life-threatening complications develop [20].

Despite these clear advantages, the practice of performing liver biopsy during bariatric surgery remains controversial [21, 22]. Concerns center primarily around the potential increased risk of operative complications in an already high-risk patient population, particularly bleeding-related complications given the vascular nature of liver tissue. While single-institution studies have investigated the safety and feasibility of intraoperative liver biopsy during BS [23–26], comprehensive analysis using large-scale national databases has been limited [16]. To address this knowledge gap, we aimed to evaluate the 30-day rate of serious complications and mortality associated with concomitant liver biopsy during bariatric surgery using the MBSAQIP database.

## Materials and Methods

### Study Design and Population

This is a retrospective cohort study of prospectively collected data. Patients included in this study were categorized into two distinct cohorts: those undergoing bariatric surgery with concomitant liver biopsy (CLB) and those without liver biopsy. The primary outcomes were to evaluate the 30-day rate of serious complications and mortality following bariatric surgery with intraoperative liver biopsy compared to those without liver biopsy. Secondary analyses were performed for specific bleeding-related complications including postoperative bleeding, transfusion requirements, and reoperation.

### Ethical Approvals

This study was reviewed and deemed exempt by the Institutional Review Board (IRB) due to the anonymity of the data.

### Data Source

Data was extracted from the MBSAQIP data registry which captures information from 902 accredited American and Canadian centers. For this study, only data from 2020 to 2022 participant user file (PUF) was included. This timeframe represents the most recent complete datasets available from the MBSAQIP at the time our study was conducted, as more recent data (2023 and beyond) had not yet been released for research access. The MBSAQIP typically releases its datasets with a lag time of approximately 1–2 years to allow for data collection, validation, and preparation. This represents a more contemporary cohort compared to previously published MBSAQIP analyses of liver biopsy during bariatric surgery, which utilized data from 2015–2018 [16].The MBSAQIP prospectively collects standardized data containing pre-, intra-, and post-operative variables specific to bariatric surgery patients.

### Patient Variables

Basic demographic data including age, sex, race, and body mass index (BMI) were collected. Patient comorbidities included the following: type 2 diabetes, hypertension, GERD, chronic obstructive pulmonary disease (COPD), hyperlipidemia, chronic steroid use, chronic kidney disease, dialysis dependency, venous stasis, preoperative therapeutic anticoagulant use, and obstructive sleep apnea. Patient history included previous venous thromboembolism (VTE), myocardial infarction (MI), percutaneous coronary intervention, and major cardiac surgery. Functional status variables encompassed preoperative functional status (defined as independent, partially dependent, or fully dependent) and American Society of Anesthesiologists (ASA) Physical Status classification.

Current Procedural Terminology (CPT) codes were used to define perioperative variables – 43,775 (sleeve gastrectomy), 43,644 (Roux-en-Y gastric bypass), 47,000 (percutaneous liver biopsy), 47,001 (percutaneous liver biopsy at time of other procedure), 47,379 (unlisted laparoscopic liver procedure).

### Objectives

The primary objective of this study was to determine the rate of major complications (Clavien-Dindo grade III and IV) and mortality (Clavien-Dindo grade V) of bariatric surgery with concomitant liver biopsy. Additionally, we sought to examine whether the safety profile of concomitant liver biopsy has changed in recent years compared to earlier analyses, particularly as surgical techniques and patient selection have evolved. Major complications were defined according to the Clavien-Dindo classification system, with grade III and IV (those requiring surgical, endoscopic, or radiological intervention) represented as a composite variable defined by one or more of the following within 30 days of surgery: anastomotic leak, postoperative bleeding, reoperation, non-operative intervention, cardiac arrest, MI, cardiopulmonary resuscitation, pneumonia, unplanned intubation, acute kidney injury, VTE, deep surgical site infection, sepsis or cerebrovascular accident. Secondary objectives included separate analyses of bleeding-related complications, specifically examining postoperative bleeding, transfusion requirements, and reoperation rates to provide more granular assessment of bleeding-related risks associated with liver biopsy. Secondary objectives included also encompassed operative times, length of stay, and specific postoperative complications including 30-day readmission rates.

### Statistical Analysis

The descriptive categorical data were expressed as percentages, and the continuous data as weighted mean ± standard deviation (SD). Univariate analyses were used to determine baseline differences between groups, using chi-squared tests for categorical data and independent sample t-tests for continuous data. Univariate logistic regression was used to compare differences between cohorts of patients with intraoperative liver biopsy and those without intraoperative liver biopsy.

A multivariable logistic regression analysis was used to identify predictive factors for serious complications and mortality within 30 days. Separate multivariable logistic regression models were constructed for bleeding-related outcomes including postoperative bleeding, transfusion requirements, and reoperation to address specific safety concerns related to liver biopsy. The available case method addressed missing data as all variables had less than 5% missingness. Patient factors and operative time were included in the model. Any variable with a p-value < 0.05 in univariate analysis was included in multivariable analysis. Variables were checked for collinearity via the variable inflation factors method. The area under the receiver operating characteristic (AUROC) curve and Brier score were used to assess validity and calibration of the multivariable models. All statistical analyses were completed using STATA 17 statistical software (StataCorp, College Station, TX, USA).

## Results

### Patient Demographics and Clinical Characteristics

Of 511,981 patients included in this study, 30,819 (6.02%) underwent bariatric surgery with CLB and 481,162 (93.98%) without CLB. The mean age was slightly higher in the CLB group (43.81 ± 11.88 vs 43.15 ± 11.74 years, *p* < 0.001). Both groups had a female predominance, though this was lower in the CLB group (80.77% vs 82.15%, *p* < 0.001). Race distribution was similar between groups, with White patients comprising the majority (54.7% vs 55.6%), followed by Black patients (30.4% vs 31.3%), and other races (14.9% vs 13.1%, *p* = 0.229).

Mean body mass index (BMI) was higher in the CLB group (45.71 ± 7.82 vs 45.02 ± 7.63 kg/m^2^, *p* < 0.001). Regarding functional status, most patients were independent in both groups (99.31% vs 99.52%), with a small proportion being partially dependent (0.67% vs 0.46%) or fully dependent (0.03% vs 0.02%, *p* < 0.001).

Patients undergoing CLB had higher rates of hypertension (28.25% vs 22.65%, *p* < 0.001), diabetes mellitus (28.25% vs 22.65%, *p* < 0.001), hyperlipidemia (25.20% vs 22.14%, *p* < 0.001), and gastroesophageal reflux disease (35.32% vs 29.59%, *p* < 0.001). The CLB group also had higher rates of obstructive sleep apnea (39.57% vs 36.77%, *p* < 0.001) and history of venous thromboembolism (2.86% vs 2.49%, *p* < 0.001). Complete baseline characteristics are presented in Table 1.

**Table 1:** Patient demographics and clinical characteristics

Characteristic	Patients with CLB (*N* = 30,819)	Patients without CLB (*N* = 481,162)	*P*-value
Demographics			
Age in years, mean ± SD	43.81 ± 11.88	43.15 ± 11.74	< 0.001
Males, *N* (%)	5,925 (19.23)	85,869 (17.85)	< 0.001
Females, *N* (%)	24,894 (80.77)	395,293 (82.15)	< 0.001
Race, *N* (%)			
White	351 (54.7)	3,189 (55.6)	0.229
Black or African-American	195 (30.4)	1,797 (31.3)	0.229
Other	96 (14.9)	748 (13.1)	0.229
Clinical Characteristics			
BMI in kg/m^2^, mean ± SD	45.71 ± 7.82	45.02 ± 7.63	< 0.001
BMI Categories, *N* (%)			
< 40	911 (2.96)	18,338 (3.81)	< 0.001
40 to 49	6,560 (21.29)	111,365 (23.15)	< 0.001
50 to 59	9,296 (30.16)	149,797 (31.13)	< 0.001
60 to 69	6,410 (20.80)	98,610 (20.49)	< 0.001
> 70	1,665 (5.40)	21,994 (4.57)	< 0.001
Functional Status, *N* (%)			
Independent	30,590 (99.31)	478,426 (99.52)	< 0.001
Partially Dependent	206 (0.67)	2,190 (0.46)	< 0.001
Fully Dependent	8 (0.03)	102 (0.02)	< 0.001
ASA Class, *N* (%)			
1–2	4,027 (13.07)	91,057 (18.95)	< 0.001
3	25,983 (84.36)	372,198 (77.47)	< 0.001
4–5	790 (2.56)	17,168 (3.57)	< 0.001
Smoker in previous year, *N* (%)	2,396 (7.77)	31,097 (6.46)	< 0.001
Comorbidities			
Hypertension, *N* (%)	8,708 (28.25)	108,934 (22.65)	< 0.001
Diabetes Mellitus, *N* (%)			
None or diet-controlled	22,112 (71.75)	372,155 (77.35)	< 0.001
Non-insulin dependent	2,585 (8.39)	29,105 (6.05)	< 0.001
Insulin-dependent	6,122 (19.86)	79,902 (16.61)	< 0.001
Hyperlipidemia, *N* (%)	7,766 (25.20)	106,546 (22.14)	< 0.001
GERD, *N* (%)	10,885 (35.32)	142,393 (29.59)	< 0.001
COPD, *N* (%)	370 (1.20)	5,320 (1.11)	0.123
OSA, *N* (%)	12,194 (39.57)	176,903 (36.77)	< 0.001
Chronic Kidney Disease, *N* (%)	161 (0.52)	2,484 (0.52)	0.884
Dialysis-dependent, *N* (%)	67 (0.22)	1,491 (0.31)	0.004
Chronic Steroids, *N* (%)	221 (2.82)	2,733 (2.05)	< 0.001
History of VTE, *N* (%)	880 (2.86)	11,969 (2.49)	< 0.001
Venous Stasis, *N* (%)	370 (1.20)	3,246 (0.67)	< 0.001
Preoperative Therapeutic Anticoagulation, *N* (%)	948 (3.08)	13,445 (2.79)	0.004
History of MI, *N* (%)	390 (1.27)	4,648 (0.97)	< 0.001
Previous PCI, *N* (%)	414 (1.34)	6,060 (1.26)	0.201
Previous Major Cardiac Surgery, *N* (%)	256 (0.83)	3,829 (0.80)	0.505

*ASA* American Society of Anesthesiologists, *BMI* Body Mass Index, *CLB* Concomitant Liver Biopsy, *COPD* Chronic Obstructive Pulmonary Disease, *GERD* Gastroesophageal Reflux Disease, *MI* Myocardial Infarction, *OSA* Obstructive Sleep Apnea, *PCI* Percutaneous Coronary Intervention, *SD* Standard Deviation, *VTE* Venous Thromboembolism

### Perioperative Outcomes

Mean operative time was significantly longer in the CLB group (96.10 ± 49.60 vs 83.59 ± 49.18 min, *p* < 0.001), representing an unadjusted difference of approximately 12.5 min. This difference in operative time should be interpreted cautiously as it may be influenced by various patient and procedural factors beyond the biopsy procedure itself. All procedures were performed laparoscopically in both groups. The median length of hospital stay was similar between groups but slightly longer in the CLB group (1.56 vs 1.54 days, *p* = 0.06). Detailed perioperative outcomes are shown in Table 2.

**Table 2:** Perioperative factors and postoperative complications

Variable	Patients with CLB (*N* = 30,819)	Patients without C:B (*N* = 481,162)	*P*-value
Perioperative Factors			
Operative time in minutes, mean ± SD	96.10 ± 49.60	83.59 ± 49.18	< 0.001
Laparoscopic approach, *N* (%)	30,819 (100.0)	481,162 (100.0)	-
Length of Stay in days, median (IQR)	1.56 (1)	1.54 (1)	0.06
Postoperative Complications			
Anastomotic Leak, *N* (%)	70 (0.23)	972 (0.20)	0.343
Postoperative Bleeding, *N* (%)	341 (1.11)	4,122 (0.86)	< 0.001
Postoperative blood transfusion, *N* (%)	251 (0.81)	3,021 (0.63)	0.014
Distribution of transfusion units, *N* (%)			
1 unit	74 (0.24)	809 (0.17)	-
2 unit	105 (0.34)	1,425 (0.30)	-
3 unit	32 (0.10)	344 (0.07)	-
4 unit	21 (0.07)	246 (0.05)	-
≥ 5 units	19 (0.06)	197 (0.04)	-
Reoperation, *N* (%)	331 (1.07)	4,407 (0.92)	0.005
Non-operative Intervention, *N* (%)	227 (0.74)	3,328 (0.69)	0.357
Readmission, *N* (%)	1,023 (3.32)	13,759 (2.86)	< 0.001
Cardiac Events, *N* (%)	40 (0.13)	434 (0.09)	0.027
Pneumonia, *N* (%)	60 (0.19)	824 (0.17)	0.337
Unplanned Intubation, *N* (%)	47 (0.15)	376 (0.08)	< 0.001
Acute Kidney Injury, *N* (%)	42 (0.14)	458 (0.10)	0.025
Venous Thromboembolism, *N* (%)	112 (0.36)	1,680 (0.35)	0.681
Deep Surgical Site Infection, *N* (%)	113 (0.37)	1,443 (0.30)	0.039
Wound Disruption, N (%)	12 (0.04)	213 (0.04)	0.854
Sepsis, *N* (%)	39 (0.13)	448 (0.09)	0.065
Cerebrovascular Accident, *N* (%)	4 (0.01)	60 (0.01)	0.938
Serious Complications, *N* (%)	971 (3.15)	13,004 (2.70)	< 0.001
Death, *N* (%)	112 (0.36)	1,680 (0.35)	0.681

*CLB* Concomitant Liver Biopsy, *IQR* Interquartile Range, *SD* Standard Deviation

### Postoperative Complications

The overall rate of major complications (Clavien-Dindo grade III and IV) was higher in the CLB group (3.15% vs 2.70%, *p* < 0.001). Specific complications that occurred more frequently in the CLB group included postoperative bleeding (1.11% vs 0.86%, *p* < 0.001), postoperative blood transfusion (0.81% vs. 0.63%, *p* < 0.001), unplanned intubation (0.15% vs 0.08%, *p* < 0.001), acute kidney injury (0.14% vs 0.10%, *p* = 0.025), and deep surgical site infections (0.37% vs 0.30%, *p* = 0.039). Further analysis of transfusion requirements showed that among patients requiring blood transfusion, the distribution of transfusion units was similar between groups, with the majority receiving 1–2 units in both CLB and non-CLB patients. The CLB group also experienced higher rates of cardiac events (0.13% vs 0.09%, *p* = 0.027) and 30-day readmission (3.32% vs 2.86%, *p* < 0.001) (Table 2).

There were no significant differences between groups in other complications, including anastomotic leak (0.23% vs 0.20%, *p* = 0.343), pneumonia (0.19% vs 0.17%, *p* = 0.337), venous thromboembolism (0.36% vs 0.35%, *p* = 0.681), wound disruption (0.04% vs 0.04%, *p* = 0.854), sepsis (0.13% vs 0.09%, *p* = 0.065), and cerebrovascular accidents (0.01% vs 0.01%, *p* = 0.938). Notably, there was no significant difference in 30-day mortality between groups (0.36% vs 0.35%, *p* = 0.681).

### Multivariable Analysis for Major Complications

Given the significant baseline differences in comorbidities between groups, multivariable analysis was essential to determine the independent effect of liver biopsy on outcomes. After adjusting for patient demographics, comorbidities, and operative factors, the most clinically significant finding was that concomitant liver biopsy was not independently associated with increased risk of major complications (Clavien-Dindo grade III and IV) (OR 0.909, 95%CI 0.795–1.040, *p* = 0.164). This adjusted analysis demonstrates that the higher unadjusted complication rates observed in the liver biopsy group are primarily attributable to differences in patient characteristics and comorbidity burden rather than the biopsy procedure itself.

The multivariable model demonstrated good calibration (Brier score 0.028) and fair discrimination (AUROC 0.66), providing strong evidence that liver biopsy can be performed safely during bariatric surgery when patient factors are appropriately considered. Complete details of all significant predictors are presented in Table 3.

**Table 3:** Significant risk factors for major complications (Clavien-Dindo Grade III and IV) on multivariable logistic regression

Risk Factor	Adjusted Odds Ratio*	95% Confidence Interval	P-value
Age (per 10 years)	1.080 (1.047–1.114)	1.047–1.114	< 0.001
Higher body mass index (per 5 kg/m^2^)	1.005 (0.984–1.026)	0.984–1.026	0.659
Diabetes (Non-insulin dependent)	1.157 (1.032–1.295)	1.032–1.295	0.012
Diabetes (Insulin dependent)	0.988 (0.907–1.077)	0.907–1.077	0.790
Concomitant liver biopsy	0.909 (0.795–1.040)	0.795–1.040	0.164
Hyperlipidemia	1.054 (0.970–1.144)	0.970–1.144	0.215
History of myocardial infarction	1.807 (1.466–2.227)	1.466–2.227	< 0.001
Operative time (per hour)	1.003 (1.002–1.003)	1.002–1.003	< 0.001
Chronic kidney disease	2.017 (1.550–2.625)	1.550–2.625	< 0.001
History of VTE	1.761 (1.521–2.038)	1.521–2.038	< 0.001
Partially dependent functional status	2.136 (1.631–2.796)	1.631–2.796	< 0.001
Totally dependent functional status	3.295 (1.133–9.582)	1.133–9.582	0.029
COPD	1.239 (0.995–1.542)	0.995–1.542	0.055
Race (Black)	1.234 (1.140–1.336)	1.140–1.336	< 0.001
Race (Other)	0.983 (0.889–1.086)	0.889–1.086	0.732

*COPD* Chronic Obstructive Pulmonary Disease, *VTE* Venous Thromboembolism. *Note*: Model adjusted for age, sex, body mass index, comorbidities, and operative time. Area under the receiver operating characteristic curve = 0.66, Brier score = 0.028

### Multivariable Analysis of 30-Day Readmission

Similarly, after comprehensive adjustment for patient factors and operative time, concomitant liver biopsy was not independently associated with increased risk of 30-day readmission (OR 1.064, 95%CI 0.936–1.209, *p* = 0.344). The model demonstrated fair discrimination (AUROC 0.65) and good calibration (Brier score 0.027). Complete details of all significant predictors for readmission are presented in Table 4.

**Table 4:** Significant risk factors for 30-day readmission on multivariable logistic regression

Risk Factor	Adjusted Odds Ratio*	95% Confidence Interval	P-value
Age (per 10 years)	0.969	0.938–1.000	0.050
Female sex	1.232	1.129–1.343	< 0.001
Higher body mass index (per 5 kg/m^2^)	1.011	0.990–1.033	0.296
Diabetes (Non-insulin dependent)	1.386	1.239–1.550	< 0.001
Diabetes (Insulin dependent)	0.974	0.891–1.064	0.557
Concomitant liver biopsy	1.064	0.936–1.209	0.344
Hyperlipidemia	1.128	1.037–1.228	0.005
History of myocardial infarction	1.606	1.280–2.015	< 0.001
Operative time (per hour)	1.003	1.003–1.004	< 0.001
History of hip surgery	1.162	1.079–1.252	< 0.001
Smoker in previous year	1.185	1.054–1.333	0.005
Roux-en-Y gastric bypass	1.847	1.717–1.987	< 0.001
Chronic kidney disease	1.841	1.394–2.430	< 0.001
History of VTE	2.070	1.797–2.386	< 0.001
Partially dependent functional status	1.571	1.156–2.135	0.004
Totally dependent functional status	1.444	0.337–6.200	0.621
COPD	1.361	1.089–1.700	0.007
Race (Black)	1.418	1.314–1.531	< 0.001
Race (Other)	0.964	0.870–1.068	0.483

*COPD* Chronic Obstructive Pulmonary Disease, *VTE* Venous Thromboembolism. *Note*: Model adjusted for age, sex, body mass index, comorbidities, and operative time. Area under the receiver operating characteristic curve = 0.65, Brier score = 0.027

### Specific Bleeding-Related Complications Analysis

Given the clinical importance of bleeding-related complications in liver biopsy procedures and to address specific safety concerns, we performed separate multivariable analyses for the most clinically relevant bleeding-related outcomes.

### Multivariable Analysis for Postoperative Bleeding

After comprehensive adjustment for patient demographics, comorbidities, and operative time, concomitant liver biopsy was independently associated with increased risk of postoperative bleeding (OR 1.203, 95%CI 1.018–1.422, *p* = 0.030). The model demonstrated good discrimination (AUROC 0.69) and excellent calibration (Brier score 0.004).

### Multivariable Analysis for Blood Transfusion

Concomitant liver biopsy was independently associated with increased risk of blood transfusion requirements (OR 1.171, 95%CI 1.028–1.334, *p* = 0.018). This finding demonstrates that liver biopsy contributes to blood loss requiring transfusion support. The model showed fair discrimination (AUROC 0.64) and good calibration (Brier score 0.006). Complete details of significant predictors for transfusion are presented in Table 5.

**Table 5:** Significant risk factors for bleeding-related complications on multivariable logistic regression

Risk Factor	Postoperative Bleeding	Blood Transfusion	Reoperation
	Adjusted OR (95% CI)	Adjusted OR (95% CI)	Adjusted OR (95% CI)
	*p*-value	*p*-value	*p*-value
Age (per 10 years)	1.055 (1.010–1.102)	1.162 (1.124–1.201)	1.121 (1.091–1.152)
	*p* = 0.017	*p* < 0.001	*p* < 0.001
Female sex	0.608 (0.547–0.676)	0.918 (0.841–1.002)	0.867 (0.806–0.933)
	*p* < 0.001	*p* = 0.056	*p* < 0.001
Higher body mass index (per 5 kg/m^2^)	0.950 (0.921–0.981)	0.939 (0.917–0.962)	0.965 (0.946–0.985)
	*p* = 0.002	*p* < 0.001	*p* < 0.001
Diabetes (Non-insulin dependent)	1.096 (0.974–1.234)	1.217 (1.114–1.331)	0.977 (0.904–1.055)
	*p* = 0.127	*p* < 0.001	*p* = 0.548
Diabetes (Insulin dependent)	0.966 (0.813–1.148)	1.207 (1.063–1.369)	0.939 (0.839–1.051)
	*p* = 0.697	*p* = 0.004	*p* = 0.276
Concomitant liver biopsy	1.203 (1.018–1.422)	1.171 (1.028–1.334)	1.016 (0.908–1.138)
	*p* = 0.030	*p* = 0.018	*p* = 0.783
Hyperlipidemia	1.216 (1.083–1.364)	1.151 (1.055–1.255)	1.053 (0.978–1.134)
	*p* = 0.001	*p* = 0.002	*p* = 0.174
History of myocardial infarction	2.201 (1.690–2.866)	1.769 (1.420–2.204)	1.276 (1.020–1.596)
	*p* < 0.001	*p* < 0.001	*p* = 0.033
Operative time (per hour)	1.003 (1.002–1.004)	1.003 (1.002–1.004)	1.003 (1.002–1.004)
	*p* < 0.001	*p* < 0.001	*p* < 0.001
Smoker in previous year	1.225 (1.032–1.455)	1.248 (1.096–1.421)	1.240 (1.112–1.382)
	*p* = 0.020	*p* = 0.001	*p* < 0.001
Roux-en-Y gastric bypass	2.793 (2.515–3.102)	1.520 (1.402–1.648)	2.354 (2.206–2.512)
	*p* < 0.001	*p* < 0.001	*p* < 0.001
Chronic kidney disease	1.304 (0.821–2.072)	2.130 (1.614–2.812)	1.653 (1.244–2.199)
	*p* = 0.260	*p* < 0.001	*p* = 0.001
History of VTE	1.971 (1.617–2.404)	1.460 (1.234–1.728)	1.440 (1.248–1.663)
	*p* < 0.001	*p* < 0.001	*p* < 0.001
Partially dependent functional status	1.905 (1.240–2.926)	2.597 (1.966–3.429)	1.755 (1.317–2.337)
	*p* = 0.003	*p* < 0.001	*p* < 0.001
Totally dependent functional status	3.947 (0.956–16.302)	5.864 (2.349–14.636)	0.803 (0.111–5.803)
	*p* = 0.058	*p* < 0.001	*p* = 0.828
COPD	1.396 (1.025–1.900)	1.326 (1.049–1.675)	1.478 (1.215–1.799)
	*p* = 0.034	*p* = 0.018	*p* < 0.001
Race (Black)	1.080 (0.956–1.221)	1.158 (1.061–1.265)	1.074 (0.996–1.157)
	*p* = 0.215	*p* = 0.001	*p* = 0.062
Race (Other)	1.166 (1.025–1.327)	0.975 (0.878–1.082)	0.890 (0.814–0.973)
	*p* = 0.019	*p* = 0.629	*p* = 0.010

Model Performance Metrics:

• Postoperative Bleeding: AUROC = 0.69, Brier score = 0.004

• Blood Transfusion: AUROC = 0.64, Brier score = 0.006

• Reoperation: AUROC = 0.67, Brier score = 0.009

*AUROC* Area Under the Receiver Operating Characteristic Curve, *CI* Confidence Interval, *COPD* Chronic Obstructive Pulmonary Disease, *OR* Odds Ratio, *VTE* Venous Thromboembolism. *Note*: Models adjusted for age, sex, body mass index, comorbidities, and operative time. Bold values indicate the concomitant liver biopsy variable, which is the primary variable of interest

### Multivariable Analysis for Reoperation

Concomitant liver biopsy was not independently associated with increased risk of reoperation (OR 1.016, 95%CI 0.908–1.138, *p* = 0.783). The model demonstrated good discrimination (AUROC 0.67) and good calibration (Brier score 0.009).

### Comparison with Earlier MBSAQIP Analyses

Our findings from the 2020–2022 MBSAQIP dataset show some notable differences compared to the 2015–2018 analysis by Clapp et al. [16]. The rate of concomitant liver biopsy appears to have increased from 3.9% to 6.02%, suggesting growing integration of this practice into contemporary bariatric surgery. The additional operative time associated with liver biopsy in our study (12.51 min) was less than previously reported (19.6 min), suggesting possible improvements in technical efficiency. While unadjusted complication rates were modestly higher in the biopsy group, our multivariable analysis, which controlled for more variables than previous studies, confirmed that liver biopsy itself does not independently increase risk when accounting for patient factors. However, our detailed bleeding-related analyses revealed specific associations with both increased postoperative bleeding (OR 1.203, *p* = 0.030) and transfusion requirements (OR 1.171, *p* = 0.018) that were not previously reported in detail, highlighting the value of examining individual bleeding-related outcomes rather than composite measures alone.

## Discussion

The relationship between obesity and liver disease has become increasingly recognized, with liver biopsy emerging as a valuable diagnostic tool during bariatric surgery. Using the largest and most contemporary nationwide database analysis to date, our study demonstrates that 6.02% of bariatric surgery patients underwent concomitant liver biopsy (CLB), reflecting a notable increase from the 3.9% reported in earlier analyses of the 2015–2018 MBSAQIP database [16]. This trend suggests growing integration of liver biopsy into contemporary bariatric practice. While initial analysis showed higher unadjusted rates of major complications (Clavien-Dindo grade III and IV) in the CLB group (3.15% vs 2.70%), our comprehensive multivariable analysis revealed that CLB itself was not independently associated with increased risk when accounting for patient factors and operative time. Similarly, despite higher unadjusted 30-day readmission rates in the CLB group (3.32% vs 2.86%), multivariable analysis demonstrated that CLB was not an independent predictor of readmission (OR 1.064, 95%CI 0.936–1.209, *p* = 0.344) after adjusting for patient characteristics. However, our detailed bleeding-related analyses revealed that CLB was independently associated with both increased postoperative bleeding (OR 1.203, 95%CI 1.018–1.422, *p* = 0.030) and transfusion requirements (OR 1.171, 95%CI 1.028–1.334, *p* = 0.018), but not with reoperation rates (OR 1.016, 95%CI 0.908–1.138, *p* = 0.783).

Our analysis yielded several clinically significant findings. The CLB group demonstrated higher rates of metabolic comorbidities, including diabetes (28.25% vs 22.65%) and hyperlipidemia (25.20% vs 22.14%), aligning with current literature showing that up to 90% of bariatric surgery patients may have underlying MASLD [27]. This patient selection pattern suggests appropriate clinical judgment in identifying those most likely to benefit from biopsy. The unadjusted increase in operative time (96.10 vs 83.59 min) represents a difference of approximately 12.5 min, which may be influenced by various patient and procedural factors beyond the biopsy itself. Nevertheless, this difference compares favorably with previous studies; Clapp et al. reported a mean additional time of 16–39.6 min for CLB procedures [16].

When compared to existing literature, our findings both validate and expand current knowledge. Our study’s larger sample size (30,819 liver biopsies compared to 21,367 in Clapp et al.) and more recent timeframe provide greater statistical power and contemporary relevance. Recent single-center studies have reported variable complication rates: Petrick et al. [28] documented a 0.23% complication rates in 434 cases, while Collins et al. [29] and Praveenraj et al. [30], reported no complications. Our larger cohort demonstrates slightly higher overall complication rates (3.15%) and importantly identifies specific bleeding-related risks that may have been underappreciated in smaller series.

The finding that concomitant liver biopsy increases both postoperative bleeding (OR 1.203) and transfusion requirements (OR 1.171) provides important clinical information extending beyond previous analyses. These risks reflect the vascular nature of liver tissue and represent absolute increases in bleeding (1.11% vs 0.86%) and transfusion rates (0.81% vs 0.63%) that surgeons must consider. Importantly, these bleeding-related complications were not accompanied by increased reoperation rates, suggesting they are generally manageable with conservative measures and blood product support when necessary.

Our 30-day readmission rate in the concomitant liver biopsy group (3.32%) falls between previously reported rates, with Jones et al. [20] reporting no readmissions in their adolescent bariatric surgery cohort, while Clapp et al. [16] documented a slightly higher rate of 3.65% in their MBSAQIP analysis. This consistency with other large database studies reinforces the generalizability of our findings and suggests that our observed readmission rates represent the expected range when CLB is performed in a variety of clinical settings.

This difference likely reflects our more comprehensive capture of complications through the MBSAQIP database and larger sample size. Importantly, our mortality rates (0.36% vs 0.35%, *p* = 0.681) were comparable to those reported in other large series of bariatric procedures without biopsy [31].

Our multivariable analysis of readmissions provides additional evidence supporting the safety of concomitant liver biopsy. We identified several independent predictors of readmission, including female sex (OR 1.232, 95%CI 1.129–1.343), non-insulin dependent diabetes (OR 1.386, 95%CI 1.239–1.550), and Roux-en-Y gastric bypass (OR 1.847, 95%CI 1.717–1.987), but CLB was not among them. These findings align with and expand upon previous work examining readmission risk factors after bariatric surgery, while providing specific evidence that CLB does not contribute to this important quality metric.

The risk factors identified through our multivariable analysis provide valuable guidance for patient selection. Chronic kidney disease, history of venous thromboembolism, and dependent functional status emerged as strong predictors of complications. These findings expand upon previous work by Barajas-Gamboa et al. [32], who identified similar risk factors but in a smaller cohort. The association of Black race with increased complications mirrors disparities documented in other aspects of bariatric care and warrants focused investigation of underlying causes and potential interventions [33].

Technical considerations in CLB have evolved significantly. While the MBSAQIP database does not capture specific technique details, our finding that operative time independently predicted complications (OR 1.003 per hour) supports the importance of surgical efficiency highlighted by Barbois et al. [4]. Recent technical reviews emphasize standardized approaches to biopsy site selection, specimen handling, and closure techniques. Our data showing minimal differences in leak rates between groups (0.23% vs 0.20%, *p* = 0.343) suggests these technical principles are being successfully implemented across participating centers.

The observed increase in concomitant liver biopsy utilization from 3.9% in 2015–2018 to 6.02% in 2020–2022 may reflect growing awareness of MASLD among bariatric surgeons, increased recognition of the potential prognostic value of histopathological diagnosis, and possibly changes in reimbursement patterns. The temporal trend coincides with the publication of updated clinical practice guidelines for MASLD management [24] and increased research interest in the relationship between metabolic surgery and liver disease. Whether this represents appropriate clinical practice evolution or potential overutilization remains an important area for future investigation. Based on our multivariable analysis, clinicians may consider concomitant liver biopsy during bariatric surgery for patients with metabolic comorbidities associated with higher MASLD risk, while being prepared for increased bleeding and transfusion requirements. Patients with diabetes mellitus, hyperlipidemia, and hypertension—who demonstrated higher rates of concomitant liver biopsy in our analysis—may derive particular benefit from histopathological assessment. However, given the demonstrated association with bleeding complications and transfusion requirements, individualized risk–benefit assessment is essential, particularly in patients with pre-existing bleeding disorders, coagulopathy, or anemia. Pre-operative optimization of hemoglobin levels, correction of coagulopathy when feasible, and availability of blood products should be considered when planning concomitant liver biopsy.

The primary strength of this analysis is that it represents the single largest study examining concomitant liver biopsy during metabolic and bariatric surgery. With over 30,000 CLB cases, the study provides substantial statistical power to detect even subtle differences in outcomes. The standardized data collection through MBSAQIP ensures consistent definitions and comprehensive capture of complications. Our multivariable analysis controlled for several potential confounders, providing more precise estimates of the independent effect of CLB. Most importantly, our separate analyses of bleeding-related complications provide granular insight into specific risks that were previously masked within composite outcomes.

Compared to previous large-scale analyses of concomitant liver biopsy during bariatric surgery, our study offers several methodological improvements. First, our analysis includes a more comprehensive multivariable model incorporating a broader range of potential confounders. Second, our separate analysis of bleeding-related complications provides a more nuanced understanding of specific risk domains that are clinically most relevant to liver biopsy procedures. Third, our use of the most recent MBSAQIP data (2020–2022) offers insights into contemporary practice patterns following the publication of updated MASLD guidelines and during a period of evolving bariatric surgical techniques.

Several limitations warrant consideration when interpreting our results. The 30-day follow-up window of the MBSAQIP prevents assessment of longer-term outcomes, particularly important given the progressive nature of liver disease. The lack of histopathological data prevents correlation of biopsy findings with complications or analysis of diagnostic yield. Additionally, we cannot account for surgeon experience or specific technical variations that might influence outcomes. The database also doesn’t capture certain relevant variables such as liver function parameters or coagulation status that could inform risk stratification.

Several important technical limitations must also be acknowledged. The MBSAQIP database does not provide information on the technical aspects of liver biopsy, including the specific technique used (such as Tru-Cut needle, scissors, or other methods), which may vary considerably across the study cohort and potentially influence outcomes. Furthermore, no data is available regarding preoperative workup such as liver ultrasound or Fibroscan results, which might inform patient selection for biopsy. Importantly, the clinical rationale behind performing liver biopsies cannot be ascertained from the database, making it impossible to determine whether biopsies were performed routinely to assess for MASLD/cirrhosis or selectively for incidentally noticed suspicious liver lesions during surgery. This lack of information on indication represents a significant limitation in interpreting the risk–benefit assessment of liver biopsy in individual patients. We acknowledge the potential for collinearity between operative time and liver biopsy variables, as liver biopsy does inherently increase operative duration. However, variance inflation factor analysis confirmed the absence of significant collinearity (all VIFs < 2), and sensitivity analysis demonstrated that removing operative time substantially reduced model discrimination (ROC approaching 0.5), supporting the inclusion of both variables. We have reported operative time per hour rather than per minute to better represent it as a surrogate for overall case complexity rather than procedure-specific duration. Importantly, the MBSAQIP database does not provide information on the clinical decision-making or specific indications that led to performing liver biopsy in individual patients, making it impossible to discern why certain patients were selected for this procedure. Additionally, we cannot determine whether bleeding complications were directly attributable to the liver biopsy site versus other aspects of the bariatric procedure, though the statistical association strongly suggests a causal relationship.

Future research directions should address these limitations. Long-term follow-up studies correlating biopsy findings with clinical outcomes could better define the prognostic value of CLB. Investigation of technical factors, including biopsy location, specimen size, and closure techniques, could help standardize best practices. Cost-effectiveness analyses comparing routine versus selective biopsy approaches would provide valuable guidance for resource allocation. Importantly, future studies should investigate strategies to minimize bleeding complications, such as optimal patient selection criteria, preoperative optimization protocols, and technical modifications that might reduce bleeding risk while preserving diagnostic yield. Liver biopsy during bariatric surgery provides crucial prognostic information beyond diagnosis, enabling identification of patients with advanced fibrosis who require enhanced surveillance, guiding treatment decisions, and stratifying patients for emerging MASH therapies. Given the high prevalence of liver disease in bariatric surgery candidates, establishing the safety of concomitant liver biopsy is essential for clinical decision-making. These findings are consistent with, but expand upon, earlier MBSAQIP analyses and provide important nuanced information regarding the bleeding-related risks of this increasingly common practice.

## Conclusions

Liver biopsy during bariatric surgery provides crucial prognostic information beyond diagnosis, enabling identification of patients with advanced fibrosis who require enhanced surveillance, guiding treatment decisions, and stratifying patients for emerging MASH therapies. After adjusting for patient factors, our analysis of this large contemporary national database demonstrates that concomitant liver biopsy during bariatric surgery does not independently increase the risk of major complications (Clavien-Dindo grade III and IV), readmission, or mortality. However, it is associated with statistically significant increases in both postoperative bleeding and transfusion requirements, though not with reoperation rates. When balancing the potential benefits of early liver disease diagnosis against risks, concomitant liver biopsy remains a reasonable option for appropriately selected patients undergoing bariatric surgery, provided clinicians are prepared for increased bleeding-related complications and blood product utilization. Careful patient selection, preoperative optimization, and availability of blood products are essential considerations when planning concomitant liver biopsy.

## Data Availability

No datasets were generated or analysed during the current study.

## Appendix 1: Variance Inflation Factor (VIF) analysis for multivariable model variables

**Table:**

Variable	VIF	1/VIF
Age (per 10 years)	1.25	0.798
Female sex	1.04	0.957
BMI (per 5 kg/m^2^)	1.07	0.937
Diabetes (Non-insulin dependent)	1.10	0.905
Diabetes (Insulin dependent)	1.14	0.877
Concomitant liver biopsy	1.01	0.994
Hyperlipidemia	1.33	0.751
History of myocardial infarction	1.03	0.973
Operative time (per hour)	1.36	0.736
Smoker	1.01	0.995
Roux-en-Y gastric bypass	1.36	0.737
Chronic kidney disease	1.02	0.981
History of VTE	1.02	0.985
Functional status (Partially dependent)	1.01	0.989
Functional status (Totally dependent)	1.00	0.999
COPD	1.03	0.974
Race (Black)	1.08	0.924
Race (Other)	1.07	0.938
Mean VIF	1.11	

All VIF values are well below the threshold of 10 that typically indicates problematic collinearity. VIF values < 2 suggest minimal collinearity concerns, supporting the inclusion of both operative time and liver biopsy variables in the model

## Appendix 2: Sensitivity analysis – model performance with and without operative time

**Table:**

Model performance comparison				
Model	Variables Included	AUROC	Brier Score	Model Performance
Full Model	All variables including operative time	0.6548	0.0267	Good discrimination and calibration
Sensitivity Model	All variables except operative time	0.5876	0.0268	Poor discrimination (approaching coin toss)

Interpretation: The sensitivity analysis demonstrates that removing operative time from the multivariable model substantially reduces discriminative ability (AUROC drops from 0.6548 to 0.5876), approaching the performance of a coin toss (AUROC 0.5). This supports the clinical and statistical importance of including operative time as a surrogate measure for case complexity, independent of liver biopsy status

Conclusion: The VIF analysis confirms absence of significant collinearity between operative time and liver biopsy variables (VIF 1.36 and 1.01 respectively), while the sensitivity analysis demonstrates that operative time contributes meaningful discriminative value to the model. Together, these analyses support the methodological decision to include both variables in the final multivariable model

## References

[1] Holterman AX, Guzman G, Fantuzzi G et al. Nonalcoholic fatty liver disease in severely obese adolescent and adult patients. Obesity (Silver Spring). 2013;21(3):591–7. 10.1002/oby.20174. 23592668 10.1002/oby.20174

[2] Doycheva I, Watt KD, Alkhouri N. Nonalcoholic fatty liver disease in adolescents and young adults: the next frontier in the epidemic. Hepatology. 2017;65(6):2100–9. 10.1002/hep.29068. 28103626 10.1002/hep.29068

[3] Corey KE, Stanley TL, Misdraji J et al. Prevalence and outcome of non-alcoholic fatty liver disease in adolescents and young adults undergoing weight loss surgery. Pediatr Obes. 2014;9(5):e91–3. 10.1111/j.2047-6310.2014.219.x. 24677740 10.1111/j.2047-6310.2014.219.x PMC4163105

[4] Barbois S, Arvieux C, Leroy V et al. Benefit-risk of intraoperative liver biopsy during bariatric surgery: review and perspectives. Surg Obes Relat Dis. 2017;13(10):1780–6. 10.1016/j.soard.2017.07.032. 28935200 10.1016/j.soard.2017.07.032

[5] Younossi ZM, Golabi P, Paik JM et al. The global epidemiology of nonalcoholic fatty liver disease (NAFLD) and nonalcoholic steatohepatitis (NASH): a systematic review. Hepatology. 2023;77(4):1335–47. 10.1097/HEP.0000000000000004. 36626630 10.1097/HEP.0000000000000004 PMC10026948

[6] Huh Y, Cho YJ, Nam GE. Recent epidemiology and risk factors of nonalcoholic fatty liver disease. J Obes Metab Syndr. 2022;31(1):17–27. 10.7570/jomes22021. 35332111 10.7570/jomes22021 PMC8987457

[7] Hillenbrand A, Kiebler B, Schwab C et al. Prevalence of non-alcoholic fatty liver disease in four different weight related patient groups: association with small bowel length and risk factors. BMC Res Notes. 2015;8: 290. 10.1186/s13104-015-1224-7. 26138508 10.1186/s13104-015-1224-7 PMC4490690

[8] Goh GB, McCullough AJ. Natural history of nonalcoholic fatty liver disease. Dig Dis Sci. 2016;61(5):1226–33. 10.1007/s10620-016-4095-4. 27003142 10.1007/s10620-016-4095-4 PMC7789914

[9] Wattacheril J, Chalasani N. Nonalcoholic fatty liver disease (NAFLD): is it really a serious condition? Hepatology. 2012;56(4):1580–4. 10.1002/hep.26031. 23038652 10.1002/hep.26031 PMC3511819

[10] Agopian VG, Kaldas FM, Hong JC et al. Liver transplantation for nonalcoholic steatohepatitis: the new epidemic. Ann Surg. 2012;256(4):624–33. 10.1097/SLA.0b013e31826b4b7e. 22964732 10.1097/SLA.0b013e31826b4b7e

[11] Kanwal F, Nelson R, Liu Y et al. Cost of care for patients with cirrhosis. Am J Gastroenterol. 2024;119(3):497–504. 10.14309/ajg.0000000000002472. 37561079 10.14309/ajg.0000000000002472

[12] De Luca M, Shikora S, Eisenberg D et al. Scientific Evidence for the Updated Guidelines on Indications for Metabolic and Bariatric Surgery (IFSO/ASMBS). Obes Surg. 2024;34(11):3963–4096. 10.1007/s11695-024-07370-7. 10.1007/s11695-024-07370-7 PMC11541402 39320627

[13] Bonner K, Heimbach JK. Obesity management in the liver transplant recipient: the role of bariatric surgery. Curr Opin Organ Transplant. 2018;23(2):244–9. 10.1097/MOT.0000000000000513. 29465440 10.1097/MOT.0000000000000513

[14] Tseng J, Korman J, Noureddin M et al. Routine versus selective liver biopsy during bariatric surgery: postoperative outcomes and preoperative predictors of NASH. Obes Surg. 2022;32(2):463–71. 10.1007/s11695-021-05797-w. 34816355 10.1007/s11695-021-05797-w

[15] Ratziu V, Goodman Z, Sanyal A. Current efforts and trends in the treatment of NASH. J Hepatol. 2015;62(1 Suppl):S65–75. 10.1016/j.jhep.2015.02.041. 25920092 10.1016/j.jhep.2015.02.041

[16] Clapp B, Dodoo C, Kim J et al. Safety of liver biopsy at the time of bariatric surgery: an analysis of the MBSAQIP database. Surg Endosc. 2022;36(1):413–21. 10.1007/s00464-021-08297-1. 33483847 10.1007/s00464-021-08297-1

[17] Neuberger J, Patel J, Caldwell H et al. Guidelines on the use of liver biopsy in clinical practice from the British Society of Gastroenterology, the Royal College of Radiologists and the Royal College of Pathology. Gut. 2020;69(8):1382–403. 10.1136/gutjnl-2020-321299. 32467090 10.1136/gutjnl-2020-321299 PMC7398479

[18] Dolce CJ, Russo M, Keller JE et al. Does liver appearance predict histopathologic findings: prospective analysis of routine liver biopsies during bariatric surgery. Surg Obes Relat Dis. 2009;5(3):323–8. 10.1016/j.soard.2008.12.008. 19356994 10.1016/j.soard.2008.12.008

[19] Lee YH, Cho Y, Lee BW, Park CY, Lee DH, Cha BS, et al. Nonalcoholic fatty liver disease in diabetes. Part I: epidemiology and diagnosis. Diabetes Metab J. 2019;43(1):31–45. 10.4093/dmj.2019.0011. Erratum in: Diabetes Metab J. 2019;43(5):731. 10.4093/dmj.2019.0188. 10.4093/dmj.2019.0011 PMC6387876 30793550

[20] Jones RE, Yeh AM, Kambham N et al. Intraoperative liver biopsy during adolescent bariatric surgery: is it really necessary? Obes Surg. 2020;30(1):69–76. 10.1007/s11695-019-04136-4. 31446562 10.1007/s11695-019-04136-4

[21] Mahawar KK, Parmar C, Graham Y et al. Routine liver biopsy during bariatric surgery: an analysis of evidence base. Obes Surg. 2016;26(1):177–81. 10.1007/s11695-015-1916-z. 26428254 10.1007/s11695-015-1916-z

[22] Spengler EK, Loomba R. Recommendations for diagnosis, referral for liver biopsy, and treatment of nonalcoholic fatty liver disease and nonalcoholic steatohepatitis. Mayo Clin Proc. 2015;90(9):1233–46. 10.1016/j.mayocp.2015.06.013. 26219858 10.1016/j.mayocp.2015.06.013 PMC4567478

[23] Verna EC. Liver biopsy at the time of bariatric surgery: a benefit for patients and the medical community. Semin Liver Dis. 2014;34(1):1–6. 10.1055/s-0034-1371549. 24782252 10.1055/s-0034-1371549

[24] Bower G, Toma T, Harling L, et al. Bariatric surgery and non-alcoholic fatty liver disease: a systematic review of liver biochemistry and histology. Obes Surg. 2015;25(12):2280–9. 10.1007/s11695-015-1691-x. 25917981 10.1007/s11695-015-1691-x

[25] Wei L, Li M, Zeng N, et al. Bariatric surgery for non-alcoholic fatty liver disease in individuals with obesity (Base-NAFLD): protocol of a prospective multicenter observational follow-up study. BMC Surg. 2021;21(1):298. 10.1186/s12893-021-01296-y. 34167531 10.1186/s12893-021-01296-y PMC8223375

[26] Younossi ZM, Koenig AB, Abdelatif D et al. Global epidemiology of nonalcoholic fatty liver disease—meta-analytic assessment of prevalence, incidence, and outcomes. Hepatology. 2016;64(1):73–84. 10.1002/hep.28431. 26707365 10.1002/hep.28431

[27] Bedossa P, FLIP Pathology Consortium. Utility and appropriateness of the fatty liver inhibition of progression (FLIP) algorithm and steatosis, activity, and fibrosis (SAF) score in the evaluation of biopsies of nonalcoholic fatty liver disease. Hepatology. 2014;60(2):565–75. 10.1002/hep.27173. 24753132 10.1002/hep.27173

[28] Petrick A, Benotti P, Wood GC et al. Utility of ultrasound, transaminases, and visual inspection to assess nonalcoholic fatty liver disease in bariatric surgery patients. Obes Surg. 2015;25(12):2368–75. 10.1007/s11695-015-1707-6. 26003548 10.1007/s11695-015-1707-6 PMC4917009

[29] Collins H, Beban G, Windsor J et al. Safety and utility of liver biopsy during bariatric surgery in the New Zealand setting. Obes Surg. 2020;30(1):313–8. 10.1007/s11695-019-04161-3. 31482482 10.1007/s11695-019-04161-3

[30] Praveenraj P, Gomes RM, Kumar S et al. Prevalence and predictors of non-alcoholic fatty liver disease in morbidly obese South Indian patients undergoing bariatric surgery. Obes Surg. 2015;25(11):2078–87. 10.1007/s11695-015-1655-1. 25835982 10.1007/s11695-015-1655-1

[31] Buchwald H, Avidor Y, Braunwald E et al. Bariatric surgery: a systematic review and meta-analysis. JAMA. 2004;292(14):1724–37. 10.1001/jama.292.14.1724. 15479938 10.1001/jama.292.14.1724

[32] Barajas-Gamboa JS, Moon S, Romero-Velez G et al. Primary single anastomosis duodeno-ileal bypass with sleeve gastrectomy (SADI-S) versus sleeve gastrectomy to SADI conversions: a comparison study of prevalence and safety. Surg Endosc. 2023;37(11):8682–9. 10.1007/s00464-023-10305-5. 37500921 10.1007/s00464-023-10305-5

[33] Mocanu V, Dang JT, Switzer N et al. Sex and race predict adverse outcomes following bariatric surgery: an MBSAQIP analysis. Obes Surg. 2020;30(3):1093–101. 10.1007/s11695-020-04395-6. 31916134 10.1007/s11695-020-04395-6

